# Supplementary material for: Kidney segmentation in neck-to-knee body MRI of 40,000 UK Biobank participants
Source: Sci Rep. 2020 Dec 1;10:20963. doi: 10.1038/s41598-020-77981-4 (PMC7708493; doi:10.1038/s41598-020-77981-4)
Supplement: Supplementary file 1 — Supplementary Information [file 41598_2020_77981_MOESM1_ESM.pdf]

Supplementary Material

Title: Kidney segmentation in neck-to-knee body MRI of 40,000 UK Biobank participants

Authors: Taro Langner, Andreas Östling, Lukas Maldonis, Albin Karlsson, Daniel Olmo, Dag Lindgren, Andreas Wallin, Lowe Lundin, Robin Strand, Håkan Ahlström, Joel Kullberg

The following material provides additional detail on the experiments and results, with code samples, the trained network weights and further documentation available on GitHub: `https://github.com/tarolangner/ukb_segmentation`

Validation details

Jaccard indices for the validation experiments are given in Supplementary Table 1. More detail, including separate evaluations of left and right kidney volumes, as well as distance, are given in Supplementary Table 2 for the network and Supplementary Table 3 for human operator variability.

Supplementary Table 1. Jaccard indices for network validation and operator variability

	Network validation		Human variability	
	Main result	Single-operator	Intra-operator	Inter-operator
Jaccard index	0.916	0.917	0.927	0.852
N	64	64	5	5

Validation on N subjects for the neural network and repeat segmentation by human operators. Whereas the single-operator validation is a classical cross-validation on dataset *A*, the main result was obtained by training on samples of both datasets *A* and *B*.

Supplementary Table 2. Detailed results of 8-fold cross-validation on 64 subjects

	Main result				Single-operator			
Property	MAE	SMAPE	R^2	LoA	MAE	SMAPE	R^2	LoA
Volume, total	9.6 cm^3	3.77%	0.950	(-25.6 to 13.9 cm^3)	8.7 cm^3	3.38%	0.951	(-22.1 to 23.2 cm^3)
Volume, left	5.4 cm^3	4.09%	0.954	(-15.2 to 8.9 cm^3)	5.0 cm^3	3.66%	0.950	(-13.8 to 14.6 cm^3)
Volume, right	4.5 cm^3	6.62%	0.968	(-12.2 to 6.9 cm^3)	4.1 cm^3	6.42%	0.971	(-9.9 to 10.8 cm^3)
Distance*	0.3 mm	0.25%	1.000	(-0.8 to 0.9 mm)	0.3 mm	0.24%	1.000	(-1.0 to 0.8 mm)

Validation metrics with mean absolute error (MAE), symmetric mean absolute percentage error (SMAPE), coefficient of determination (R^2) and 95% limits of agreement (LoA). One subject with a missing right kidney was excluded from the distance measurements and causes high values for the SMAPE metric as an outlier.

Supplementary Table 3. Detailed operator variability on 5 subjects

	Intra-operator variability				Inter-operator variability			
Property	MAE	SMAPE	R^2	LoA	MAE	SMAPE	R^2	LoA
Volume, total	5.6 cm^3	2.56%	0.994	(-4.5 to 13.4 cm^3)	27.0 cm^3	10.23%	0.839	(-59.0 to 5.0 cm^3)
Volume, left	2.0 cm^3	1.93%	0.996	(-2.1 to 5.4 cm^3)	13.0 cm^3	10.28%	0.870	(-25.1 to -0.92 cm^3)
Volume, right	3.6 cm^3	3.15%	0.990	(-2.8 to 8.3 cm^3)	14.0 cm^3	10.18%	0.798	(-34.4 to 6.3 cm^3)
Distance	0.2 mm	0.14%	1.000	(-0.6 to 0.4 mm)	0.4 mm	0.29%	1.000	(0.1 to 0.7 mm)

Blinded repeat segmentation by the operator of dataset *A* resulted in the listed intra-operator variability. The inter-operator variability was determined by averaging the measurements per subject for the two operators of dataset *B* and comparing the result to those of operator *A*.

Algorithmic quality ratings

This section describes the individual algorithmic quality ratings in detail. Their distribution in the inference run is shown in Fig. 5 of the main document. Supplementary Fig. 1 shows examples of characteristic failure cases.

When the segmentation yields more than just two connected components, the sum of left and right individual volume may be exceeded by the measured total segmented volume. The share of this superfluous *scrap volume* is an important indicator for potential segmentation failure, as spurious segmentation of disjunct areas is a common failure case of many neural network architectures. This effect can also occur in anomalous cases with severe cystic formations that fragment the kidney tissue. In turn, less than two connected components can occur when only one kidney is segmented. This, too, may not necessarily reflect a failure of the network, as prior surgical removal and genetic variations such as fused horseshoe kidneys may arguably lead to only one volume of healthy kidney tissue existing in the image. Anomaly of the imaging process due to undetected water-fat swaps, motion, and non-standard image contrast also affect this behaviour.

In image fusion, the *segmentation fusion cost* is defined as the sum of absolute differences in the overlap of two adjacent stations, based on their voxel-wise binary labels. The *image fusion cost* uses the same term on the voxel-wise water signal intensities and normalizes the sum with the range of existing intensity values to take into account variations in contrast. High values of these terms indicate that the anatomy in both stations does not line up smoothly due to motion or other artefacts.

Another potential problem is misalignment of the subject, so that the kidneys are not fully contained in the second and third imaging station. The *positioning cost* rating quantifies the offset of the kidney centres of mass from the centre line of the image along the longitudinal axis. High values consequently identify those subjects with segmented kidney volume that is placed too high or too low on the bed, potentially reaching beyond the field of view.

The *segmentation smoothness* of the fused binary labels is then rated by summing up the absolute differences between the segmentation volume to a copy of itself, which is shifted by one voxel along the longitudinal axis. Any sudden change in segmentation between adjacent axial slices is thereby penalized, and high values indicate spurious gaps or islands created by the network.

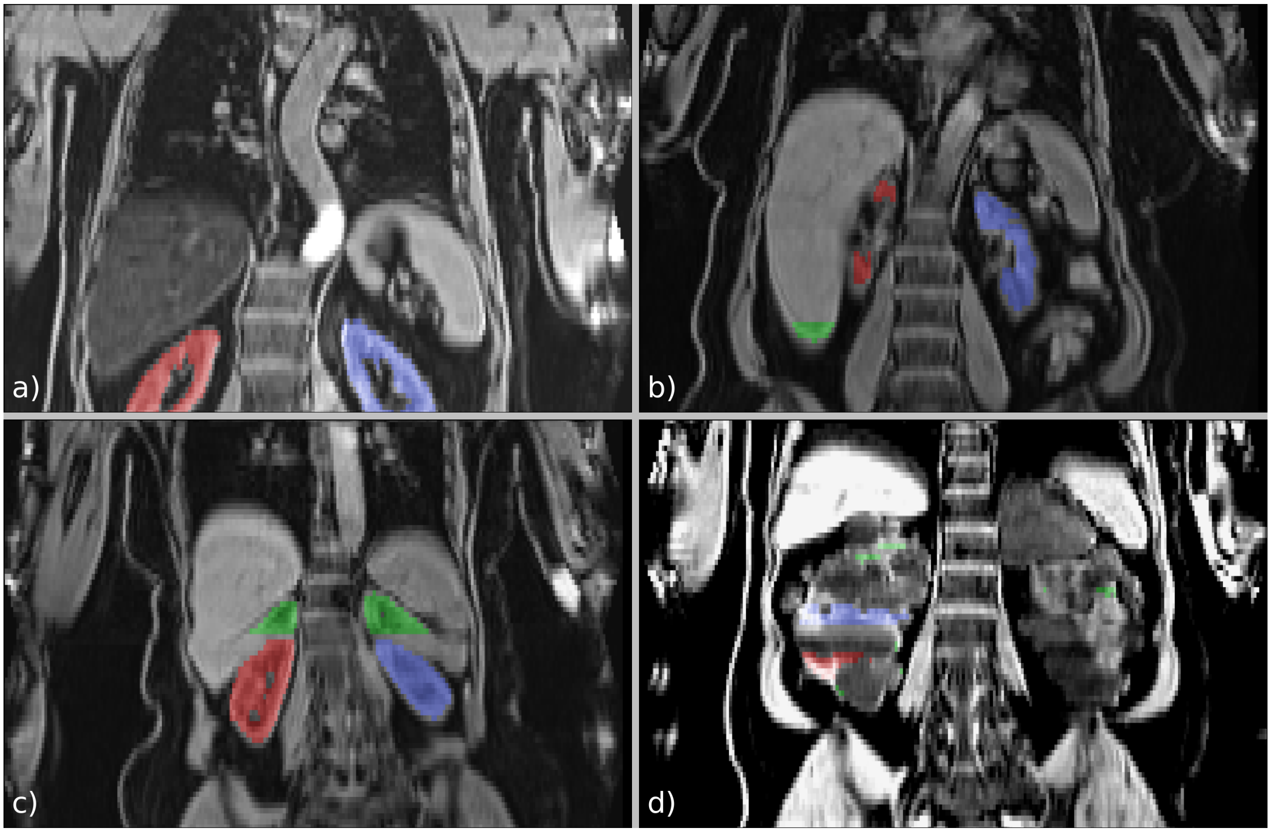

Supplementary Figure 1. Four characteristic failure cases with segmented right (red), left (blue), and disjunct scrap (green) kidney labels overlaid in coronal view for: an outlier in vertical location (a), genuine segmentation failure, where the network marked parts of the liver (b), failure due to motion during imaging, despite an arguably correct segmentation (c), and severe fragmentation of healthy tissue by cysts (d).

Kidney offsets

The left and right kidney are identified as the two largest connected components in the segmented volume. Based on the centre of mass, their relative position in the human body can be described, with euclidean distance values given in Supplementary Table 4.

Supplementary Table 4. Inferred UK Biobank parenchymal kidney distance in mm

Property	mean ± SD	[min, max]	median	(10%, 90%)
**Distance**				
male	153 ± 15	[0, 211]	153	(137, 171)
female	127 ± 12	[0, 215]	127	(114, 142)

Male (N=17,846) and female (N=19,622) distance between left and right centres of mass of parenchymal kidney volume. SD denotes the standard deviation.
